# Transboundary spread of peste des petits ruminants virus in western China: A prediction model

**DOI:** 10.1371/journal.pone.0257898

**Published:** 2021-09-23

**Authors:** Shan Gao, GuoYong Xu, Zan Zeng, JiaNing Lv, LiYa Huang, HaoNing Wang, XiaoLong Wang

**Affiliations:** 1 College of Wildlife and Protected Area, Northeast Forestry University, Harbin, Heilongjiang province, P. R. China; 2 Key Laboratory of Wildlife diseases and Biosecurity Management of Heilongjiang Province, Harbin, Heilongjiang province, The People’s Republic of China; 3 The Second Geomatics Cartography Institute of National Administration of Ministry of Natural Resources, Harbin, Heilongjiang province, P. R. China; 4 Changbai Mountain Academy of Sciences, Antu, Jilin province, P. R. China; 5 School of Geography and Tourism, Harbin University, Harbin, Heilongjiang province, The People’s Republic of China; University of Lincoln, UNITED KINGDOM

## Abstract

In pan Pamir Plateau countries, Peste des petits ruminants (PPR) has brought huge losses to the livestock industry and threaten the endangered wildlife. In unknown regions, revealing PPRV transmission among countries is the premise of effective prevention and control, therefore calls for quantified monitoring on disease communication among countries. In this paper, a MaxEnt model was built for the first time to predict the PPR risk within the research area. The least cost path (LCP) for PPR transboundary communication were calculated and referred to as the maximum available paths (MAP). The results show that there are many places with high-risk in the research area, and the domestic risk in China is lower than that in foreign countries and is mainly determined by human activities. Five LCPs representing corridors among Kazakhstan, Tajikistan, Pakistan, India and China were obtained. This study proves for the first time that there is the possibility of cross-border transmission of diseases by wild and domestic animals. In the future, it will play an important role in monitoring the PPR epidemic and blocking-up its cross-border transmission.

## Introduction

Peste des petits ruminants (PPR) is an acute, contagious disease caused by the infection of peste des petits ruminants virus (PPRV) belonging to the genus of morbilliviruses [1]. PPR is clinically characterized by sudden depression, high fever, anorexia, oculonasal discharges, oral necrotizing and erosive ulcers, stomatitis, gastroenteritis, diarrhea, and bronchopneumonia [2]. Small ruminants are infected by contact with the secretions or feces of infected animals or sharing via pasture, water or salt bodies [3]. In infection, morbidity and case fatality rate remain high and the maximum is 100% [4]. Both domestic small ruminants and wild ungulates are vulnerable to PPRV. *Capra ibex*, *Gazella subgutturosa*, *Capra ibex*, *Pseudois nayaur* and *Ovis ammond* are natural suspectible hosts [5]. In addition, unusual hosts such as camel, cattle, buffalo, dogs, Asiatic lion and pigs have been reported [6]. Notably, due to the limited data from wildlife, the role of wildlife in PPR epidemiology is still not clearly understood [7].

PPR was first discovered in the early 1940s in Cote d’I voire and then spread to Africa and parts of Asia [8]. To date, around 70 countries have either reported infection to the OIE or are suspected of being infected [9]. Over 60% of these countries are located in Africa. The current prevention and control measures for PPR are engaged according to the PPRV infection history in the local area. In an endemic area, a live-attenuated vaccine is applied to stimulate herd immunity in the livestock [10]. In previously PPR-free areas, the slaughter of infected animals, sustainable sanitation of the living environment, prohibition of animal transportation and quarantine are commonly adapted [11]. Although many measures have been taken in order to prevent the dissemination of PPR, little success has been achieved [12]. Over 63% of the domestic small ruminants in the world remain under the threats of PPR, while the risk to wildlife remains unknown [13]. In the affected countries or regions, the livestock industry was destroyed or severely damaged by the PPR, which further leads to an economic recession and even threaten the food security of the local society [14]. As a result, PPR has attracted the attention of international organizations and relevant national authorities and is listed as a transboundary animal disease which needs to be controlled and eradicated by Food and Agriculture Organization of the United Nations (FAO) and World Organization for Animal Health (OIE) [15].

The first PPR epidemic happened in the Ngari region of Tibet, China, in 2007 resulting in the death of 5751 sheep [16]. In October 2007, wild bharal (*Pseudois nayaur*) were confirmed infected in Ge’gyai County. Isolates from bharals (*Pseudois nayaur*) and domestic small ruminants were closely related [17]. In December 2013, PPR emerged in Xinjiang, China and rapidly spread to the rest of China by the first half of 2014 [18]. China isolates obtained in these two epidemics are closely related to that from bordering countries belonging to lineage IV, but different branches [19]. Interestingly, the PPRV strain identified in Xinjiang in 2013–2014 displayed higher genetic similarity with the virus strains from Pakistan [20] and Tajikistan than the strain identified in Tibet in 2007 [21]. Taken together, while the details in PPRV transmission to China remain to be fully revealed, it seems that the two PPR outbreaks in China might be independent events caused by virus transmission from the bordering endemic countries.

In the research area, some wild ruminant species are threatened by PPRV, domestic small ruminants most likely spread the virus to wild small ruminants. Subsequently, infected wildlife continue to transmit the virus to other susceptible wildlife. Most of the information about PPRV comes from domestic animals, and the research on wild animals is relatively limited [22]. The huge gap between the epidemiological research of domestic and wild ruminants makes the situation even more complicated for disease eradication. As such, the prediction of the model may become an effective means of disease prevention and control. Studies have shown that unrestricted movement of animals within a country and across borders is considered a major cause of the spread of infectious diseases [23]. A resistance coefficient is used to express the ability of a species to pass through a specific landscape unit or the suitability index of the landscape unit for a species [24]. In the process of passing through a specific environment, if the energy and time consumed by an individual are small and the mortality rate is low, the environmental resistance is low [25]. Therefore, the possibility of an individual passing through an area is high. The landscape unit in this scenario is considered to be a corridor or migration path [26]. We hypothesized that there are natural migration paths for wild and domestic ruminants near the western border of China (N 29°54’ - 44°32’), which may serve as a means of cross-border transmission of PPRV due to grassland contamination from many species sharing the same food source (grass) [27]. First, we predicted the distribution of PPRV on both sides of the border using MaxEnt and the migration paths among different PPRV-contaminated regions using an LCP model. This revealed the potential transboundary spread of PPRV, which could aid in reducing such transmission between countries at the border.

## Materials and methods

### Research area

**Fig 1:**
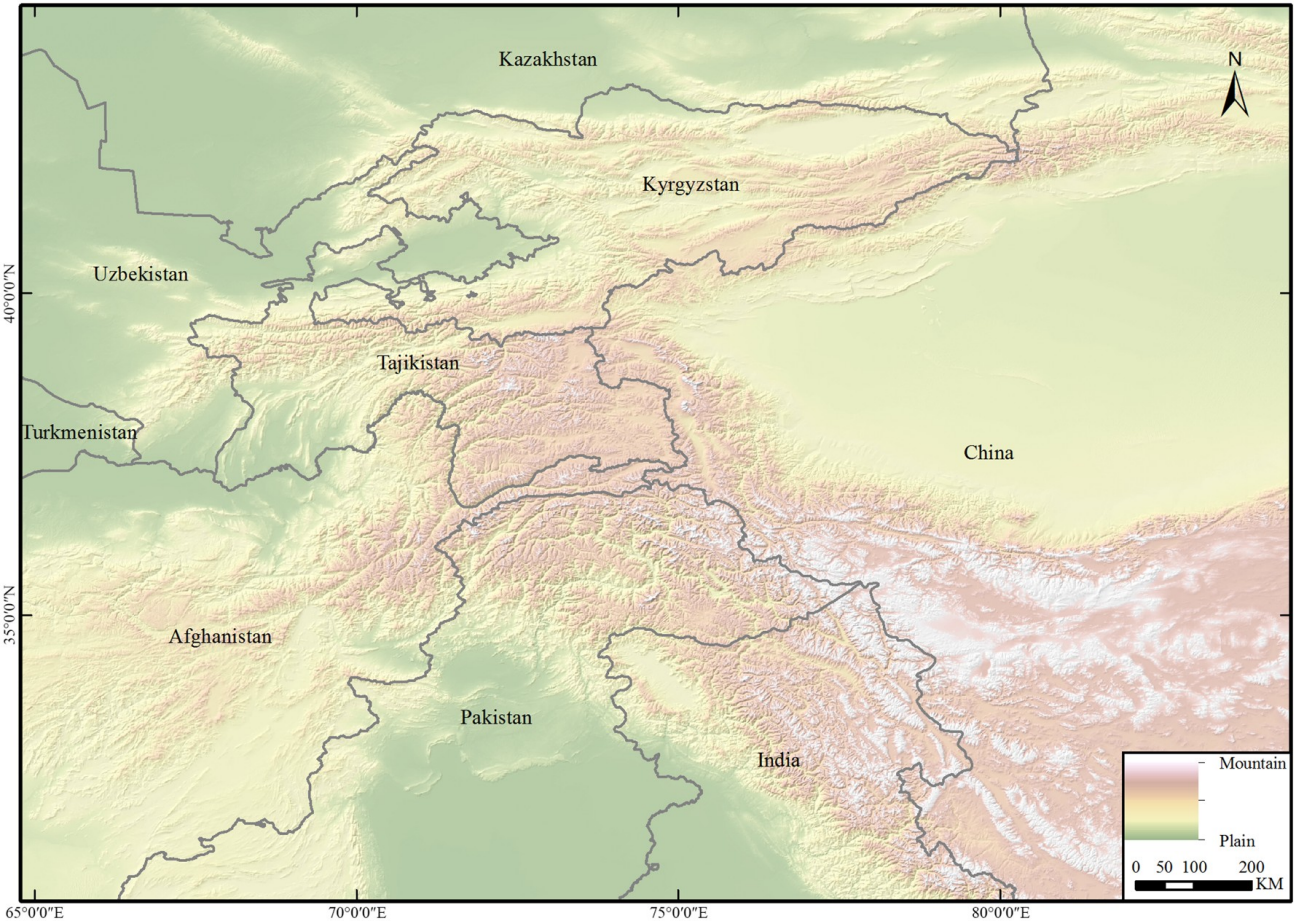
The research area. The elevation depicted by the digital elevation model (DEM). DEM was obtained from USGS Earth Explorer (https://earthexplorer.usgs.gov); the boundary was obtained from Natural Earth (http://www.naturalearthdata.com/), which is a schematic line illustrating the relative position of each country and should not be re-used or misinterpreted for any political reason.

The study area (N 29°54’ - 44°32’) mainly includes Xinjiang and Tibet in China, the western border, and part of the Chinese borders with India Pakistan, India, Pakistan, Afghanistan, Tajikistan, Kyrgyzstan and Kazakhstan (Fig 1). The Pamir Plateau and its mountain range are located in the southeast of Central Asia and the west end of China. The Pamir Plateau also straddles southern Tajikistan and northern Afghanistan. In this area, the Kunlun Mountains, Karakoram Mountains, Hindu Kush Mountains, and Tianshan Mountains join, to cover an area of approximately 1×10^5^ square kilometers. The Pamir Plateau is composed of several groups of mountains and wide valleys and basins between mountains, with an average elevation of more than 5000 m. It has a strong continental alpine climate with an extremely cold and long winter, especially in the eastern Pamirs. There is a clear difference between the east and west Pamir Plateau. The west Pamir Plateau is a typical high mountain plateau with high absolute and relative heights and a rocky terrain. The abundant rainfall enables the development of dense river networks and vegetation. In contrast, the absolute and relative heights of the eastern Pamir Plateau are smaller. The 8041 square kilometers of land in the east Pamir Plateau is mainly covered by wide valleys and 1085 glaciers. It is also the breeding ground for many small wild ruminants. At the same time, wild ruminants can migrate across the border freely. Furthermore, domestic animal rearing is common in this area, particularly the rearing of sheep and goats by nomadic communities.

### Research data

We obtained the PPR outbreaks points (n = 396) from literature and the OIE reports. To determine the influence of the environmental variables on the PPR distribution, we considered four fundamental environmental predictor categories (Table 1), namely climate, terrain, vegetation and human impact [28]. All spatial data preprocessing and calculations were done with standard operations in ArcGIS 10.2 and projected in UTM-WGS-1984. Where necessary we resampled to 30 arc-seconds.

**Table 1:** Data layer and source, raster/vector, value range/categories (number of subcategories in brackets) and specification of the unit of measurement/impact (proxy).

Layer	Source	Value/categories	Variable/proxy
**Climate** ^a^			
Monthly P	CHELSA	0 to 275 mm/month	Precipitation
Monthly mean T	CHELSA	-32.6 to 37.3°C	Mean Temperature
Monthly min T	CHELSA	-37.3 to 30.5°C	Minimum Temperature
Monthly max T	CHELSA	-27.9 to 43.6°C	Maximum Temperature
Bioclimatic	CHELSA		Annual trends, seasonality, extreme or limiting environmental variables
ISR-spring	ASTER-GDEM	8.1 to 84.2 wh/m^2^	Topo-climate
ISR-summer	ASTER-GDEM	12.1 to 97.3 wh/m^2^	Topo-climate
ISR-autumn	ASTER-GDEM	3.1 to 60 wh/m^2^	Topo-climate
ISR-winter	ASTER-GDEM	2.8 to 92.2 wh/m^2^	Topo-climate
**Terrain**			
Elevation	ASTER-GDEM	-10 to 8844 m a.s.l	Climbing distance
Slope angle	ASTER-GDEM	0 to 88.2°	Climbing effort
Distance to river	ASTER-GDEM	0 to 410.7 km	Water source
**Human impact**			
Human population	WorldPop ^b^	0 to 14229 persons/km^2^	Human-Animal interaction
**Vegetation** ^c^			
Land cover	ESA	Cropland (3), Herbaceous, Tree (9), Shrubland (3), Grassland, Urban areas, Bare areas (2), Mosaic shrub & herbaceous cover, Water bodies, Permanent snow, and ice	Animal food and refuge

^a^ T = temperature; P = precipitation; Source: CHELSA 1.2 (http://chelsa-climate.org/) at 30 arc-second resolution; ISR = Incoming Solar Radiation.

^b^ Source: https://www.worldpop.org/.

^c^Source: Land cover map (https://maps.elie.ucl.ac.be/CCI/viewer/); the number of subcategories in parentheses.

### PPR spatial distribution model

In the construction of the model, due to the great difference in the terrain of the study area, in order to overcome the problem that the model is not robust enough, the regions with significant differences in elevation are treated separately [29]. The low-elevation model (Model 1) and a high-elevation model (Model 2) were constructed for regions below and above 1500 m, respectively, according to the elevation standard of highland climate [30]. The spatial autocorrelation was minimized by filtering all recorded PPR locations using the SDM Toolbox v1.1c in ArcGIS 10.6 [31]. Filtering was performed by limiting the minimum distance between each pair of points. Multicollinearity was reduced for both the climate and non-climate predictors. First, major predictors were selected. Principal component analysis (PCA) was carried out using SPSS 22.0 [32]. The variables with eigenvalues larger than 1.0 and the scree plot criterion or ’broken stick’ stopping rule for PCA in item-level factoring were adopted [32]. Suppression of unnecessary loading and rotation of factor pattern of variables was used to retain predictors for subsequent analysis in MaxEnt [33]. Next, the filtered PPR locations and predictors were used as input data for the MaxEnt model. The area under the ROC curve (AUC) is selected to guarantee the robustness of the MaxEnt model [34]. We divided the selected presence records into 70% training and 30% testing portions to build and validate the models based on 10 bootstrap replicates [35]. For the remaining parameters, we kept the default settings in the pilot study. In addition, the Jackknife test and the variable response curves were selected to identify the relative contribution of predictor variables to the model. The least contributing predictors of the non-collinear variables were eliminated stepwise and eliminated predictor variables with a high standard deviation (SD) based on visual observation of the response curves [36]. Finally, variance inflation factor (VIF) analysis was conducted to evaluate the multicollinearity among predictors after the reduction. A VIF value below 10 indicates low and acceptable multicollinearity [37, 38]. Predicted PPR risk maps obtained by models 1 and 2 were overlaid using the fuzzy overlay to construct the final PPR risk map [31].

#### Prediction of the maximum available transboundary paths (MAPs)

LCP model can predict migration routes for ruminants by integrating geographic and behavioral information to comprehensively predict the potential transboundary path of the animals. We combined the species and habitats of wildlife in the study area and created a cost surface for PPR-infected small ruminants dispersal in the Transmission region using reclassified land cover and elevation as cost factors. The reclassification followed the Jenks natural breaks method [39]. We determine the cost values of the land cover and elevation according to small ruminants movement preference. Factors such as elevations between -10 to 5200m, forest, shrubland, Mosaic herbaceous, Grassland were assigned with the lowest cost value (= 1) [40]. Raster cells with elevations > 5200 m, bare areas, Permanent snow and ice, water bodies, etc. were assigned the highest cost value (= 9). The land cover and elevation of the reported PPR cases were each evaluated with a scale of 1 (available) to 9 (unavailable) [41]. The PPR outbreak points into China and abroad in the study area are analyzed by clustering using ArcGIS 10.2m (Grouping analysis), combined with the cost surface, and LCP is constructed pair-wised (between clusters) of points. The final MAPs were obtained by excluding paths with a starting point far away from the border or incorporated into the other most convenient paths. We called the transboundary LCPs for the transmission of PPR by small ruminants in the pan Pamir Plateau the maximum available paths (MAPs).

## Results

### Results of PPR spatial distribution models

**Fig 2:**
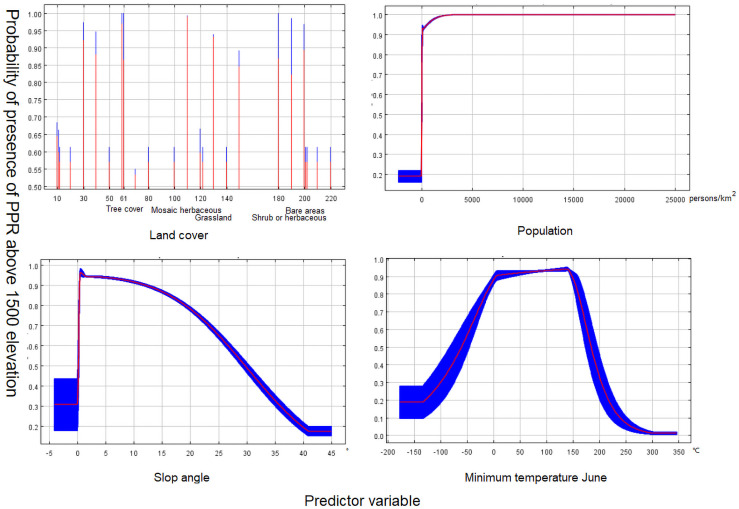
The response curves of the model (≤1500 m). The curves show the mean response (red) and the mean standard deviation (blue).

In areas above or equal to 1500 meters, 99 geographical locations of previously reported PPR cases were left by 30 km rarefying. After PCA and MaxEnt filtering, the minimum temperature of June, vegetation, population density and slope were adopted for the construction of the final model. No multicollinearity was detected with VIF values of 0 to 2 (<10) between predictors. For validation of the model, AUC = 0.825, SD = 0.027, indicating a good prediction, the response curves of the different predictors are shown in Fig 2, and the relative contributions of each predictor are shown in Table 2 (left).

**Table 2:** Estimates of relative contributions of the predictor variables.

**Above 1500m elevation**		
**variant**	**Contribution%**	**Permutation importance**
Population Density	32.2	30.3
Min T June	28.4	45.9
Slop angle	22.7	10.2
Land Cover	16.6	13.6
**Below 1500m elevation**		
**variant**	**Contribution%**	**Permutation importance**
Population Density	46.8	21.9
Mean T Sep	38.8	69.7
Land Cover	10.7	5
Slop angle	3.7	3.3

**Fig 3:**
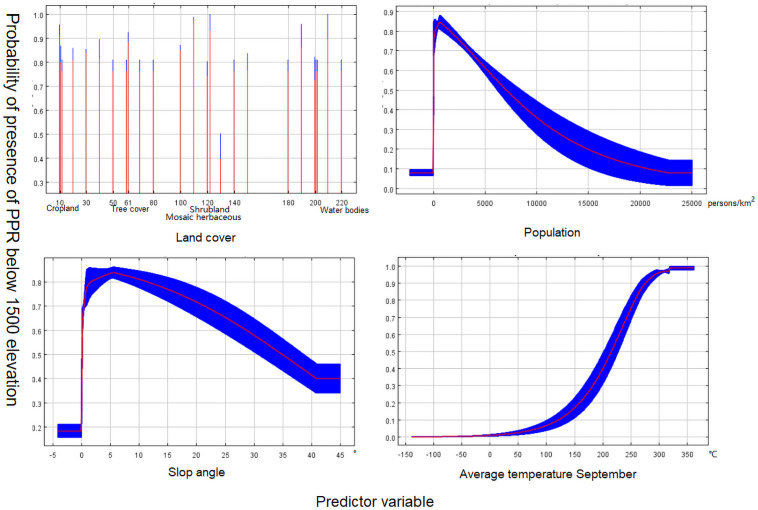
The response curves of the model (>1500 m). The curves show the mean response (red) and the mean standard deviation (blue).

In areas below 1500 meters, 81 geographical locations of PPR cases were left by 40 km rarefying. After PCA and MaxEnt filtering, the mean temperature of September, vegetation, population density and slope were adopted for the construction of the final model. No multicollinearity was detected with VIF values of 0 to 2 (<10) between predictors. For validation of the model, AUC = 0.890, SD = 0.005, indicating a good prediction, the response curves of the different predictors are shown in Fig 3, and the relative contributions of each predictor are shown in Table 2 (left).

**Fig 4:**
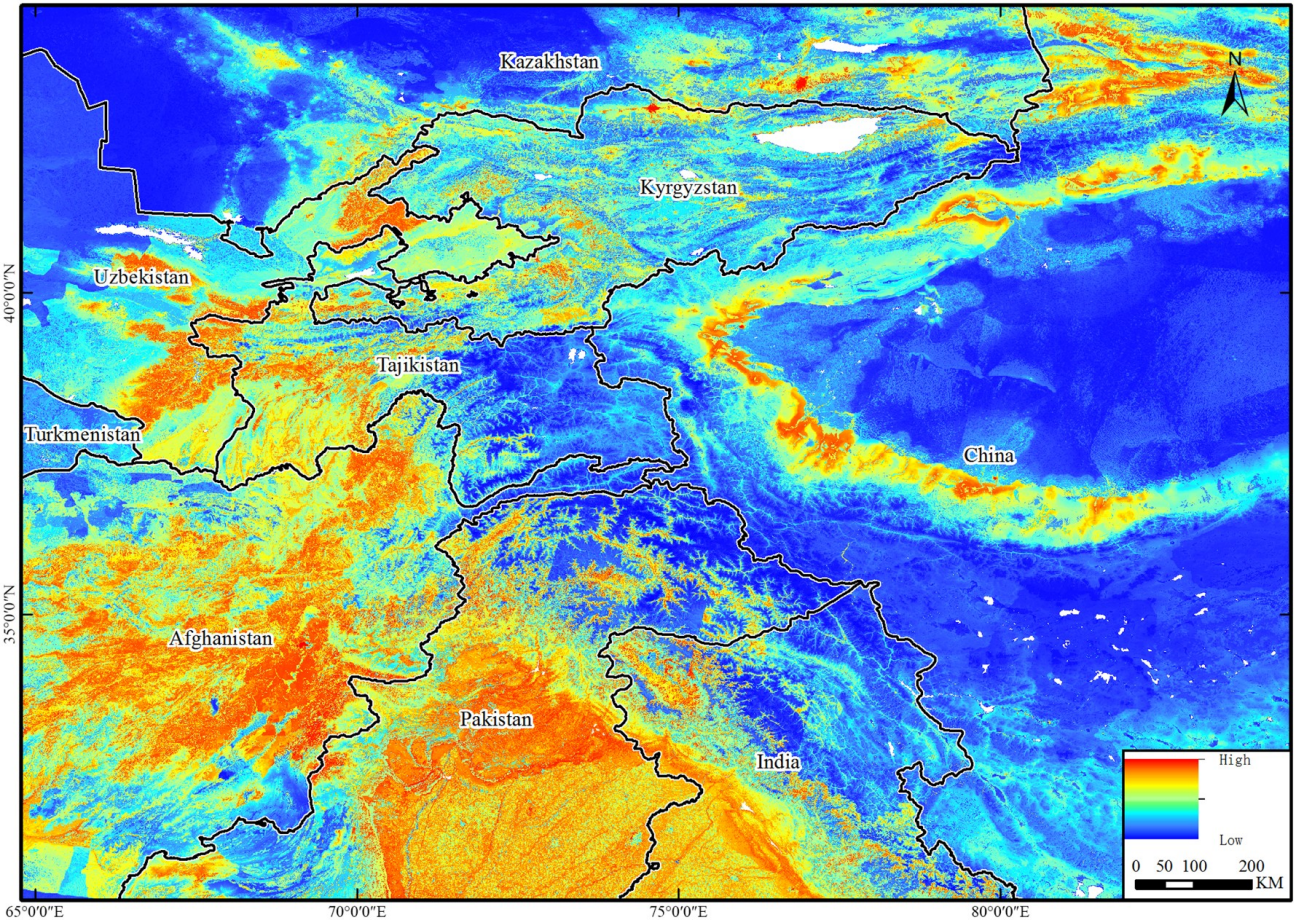
PPR high-risk areas were predicted by the MaxEnt model. This map was made in ArcGIS 10.6 using the resulting rasters produced by MaxEnt. The boundary was obtained from Natural Earth (http://www.naturalearthdata.com/), which is a schematic line illustrating the relative position of each country and should not be re-used or misinterpreted for any political reason.

Results showed that the Pamirs Plateau and its extended mountains are high-risk areas. The countries bordering the western part of China within this region are all high-risk regions for PPR. Tibet and Xinjiang of China are surrounded by these risk areas. There are high-risk areas in China, but compared with neighboring countries, the risk is lower (Fig 4). PPR risk is regulated by anthropoid and meteorological factors.

### Results of the LCP model

**Fig 5:**
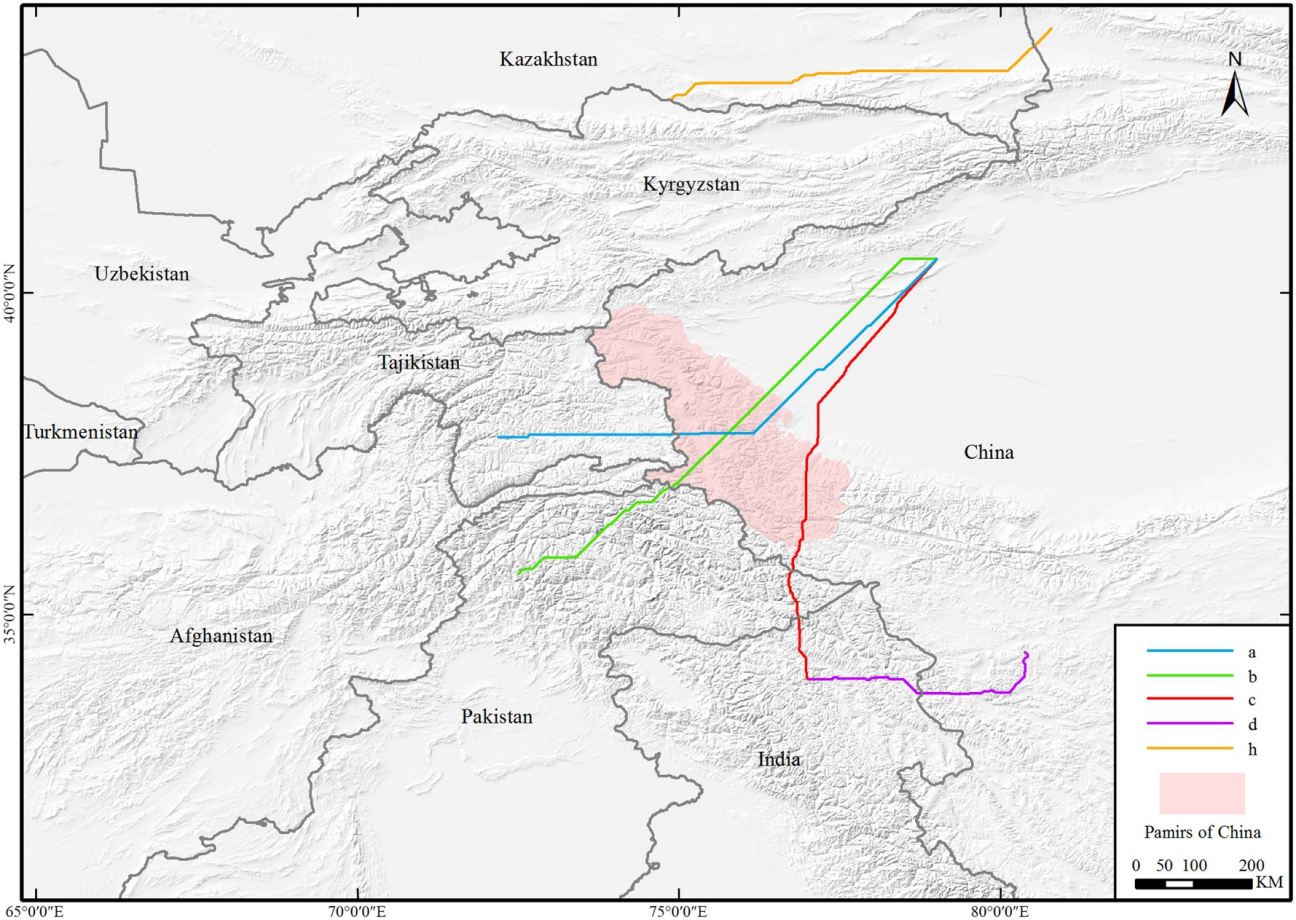
Transboundary LCPs for small ruminants. The boundary was obtained from Natural Earth (http://www.naturalearthdata.com/), which is a schematic line illustrating the relative position of each country and should not be re-used or misinterpreted for any political reason.

**Fig 6:**
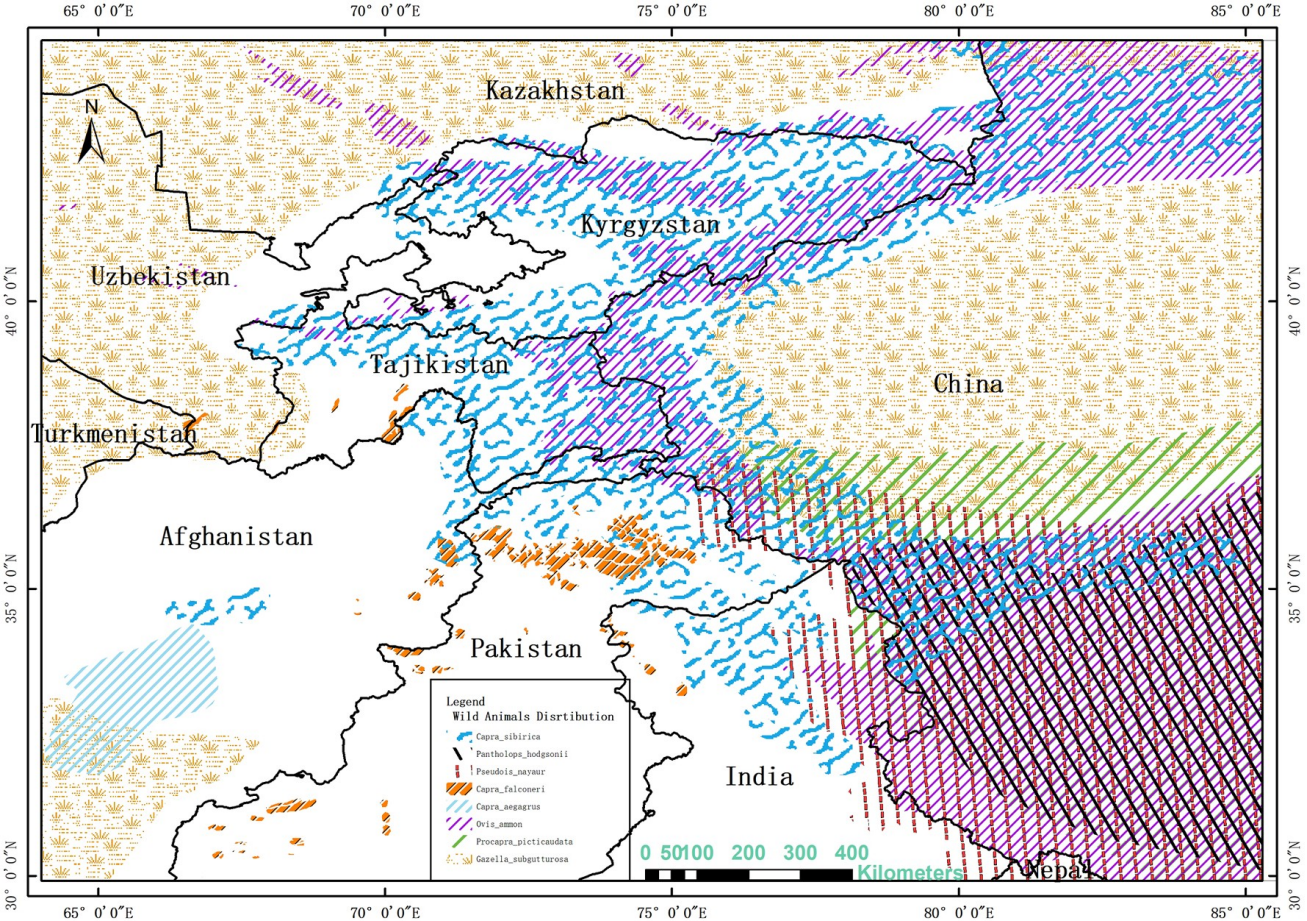
The distribution of wild ruminants. The territory range of wild small ruminants was obtained from International Union for the Conservation of Nature (IUCN) website (https://www.iucnredlist.org/). The boundary was obtained from Natural Earth (http://www.naturalearthdata.com/), which is a schematic line illustrating the relative position of each country and should not be re-used or misinterpreted for any political reason. The data used for this figure is under CCBY license and permission for its use has been obtained from the IUCN.

The LCP analysis revealed five potential transboundary paths (Fig 5) in the research area: a. Kazakhstan-Confluence of Ili River and Horgos River-Xinjiang (Huocheng county); b. Tajikistan -West Pamir Plateau-Xinjiang (Kashgarcity); c. Pakistan-West Pamir Plateau-Xinjiang (Kashgarcity); d. Kashmir-Pakistan-West Pamir Plateau-Xinjiang (Kashgarcity); e. Kashmir -Bangonlake -Tibet (Rotug county). All paths are covered by small wild ruminants (Fig 6).

## Discussion

The results show that Xinjiang and Tibet of China as well as Pakistan, Afghanistan, Kazakhstan and Kyrgyzstan were all classified as high PPR risk areas. This result is in line with the previously reported PPR situation. The risk areas are mainly driven by human factors, land cover, temperature and slope. Five LCPs representing corridors among Kazakhstan, Tajikistan, Pakistan, India and China were obtained. This study proves for the first time that there is the possibility of cross-border transmission of diseases by wild and domestic animals. In the future, it will play an important role in monitoring PPR epidemics and blocking-up its cross-border transmission.

The human population density is the most important predictor in our model as the factor with the highest contribution rate. Different association patterns of PPRV risk and population density were observed in areas above or below 1500 m. In high elevation areas, the increase in population density is correlated with a gradual increase in PPRV risk before reaching a plateau. Below 1500 m, the PPRV risk first increases and then decreases rapidly as the population density increases. This result can be explained by differences in culture and human activity among areas of different elevations. Most of the high elevation areas are pasturing areas where small ruminants, such as sheep and goats, are the most common livestock. Therefore, a high human population density is expected to be associated with a larger herd size and increased PPRV risk. In contrast, low-elevation areas mainly consist of human habitats of different sizes. Smaller towns and villages that are less urbanized may still maintain pasturing activity. Therefore, it appears that large populations of small ruminants and live sheep breeding facilitate the spread of PPRV. The highly urbanized cities in this area only keep a limited number of livestock animals, which explains the decrease in PPRV risk associated with high population density. These results show that human activities have a significant impact on the occurrence of PPRV [42].

In addition to the population density, the temperature is another important influencing factor in the constructed models. The risk of PPR displayed a positive correlation with temperature in both high and low elevation areas. While not validated in the case of PPR specifically, the effect of temperature on disease spreading has been demonstrated in multiple studies [43, 44]. However, it has also been noted that the minimum temperature of June and the mean temperature of September were used to predict the risk of PPR in high and low elevation areas respectively. This can be explained by the temperature plays a key role in disease transmission and reminds us that the prevention and control of PPR should pay attention to the change of temperature [44, 45].

Terrain factors were also analyzed in our models. In areas above 1500 m, the risk of PPRV decreased with an increase in slope, which may be related to the fact that animals prefer to inhabit areas with smaller slopes. A small slope also indicates sufficient water and food. According to the response curve of land cover, habitats with mosaic herbaceous cover trees and shrubs as well as grasslands are associated with the highest PPRV risk. Deciduous shrublands and grasslands are the main food sources of small ruminants. In addition, the trees in deciduous broad-leaved forests can provide shelter for small wild ruminants, which may become a gathering place. As a result, ruminants are more likely to spread the disease in these areas.

Five paths were predicted in this study with four starting points outsides of China and three end points located inside of China respectively. Paths a, b and c start from Tajikistan, Pakistan and India respectively, but they all use the West Pamirs to enter China. This region is characterized by abundant rainfall and vegetation, providing an ideal habitat for wild ruminants. The Chinese Taxkorgan Wildlife Nature Reserve is located in this area. The reserve is home to many rare wild animals, such as *Procapra przewalskii*, *Ovis ammon polii*, *Pseudois Nayar* and *Capra sibirica*, which can host PPRV. Moreover, mountain passes act as natural corridors between China, India, and Afghanistan. Due to ecological and environmental predisposing factors, the west Pamir Plateau is associated with a high risk of PPR transmission. More monitoring of wildlife is required in this area. Path d starts from Kashmir, passes along the bank of Pangong Tso Lake, and finally enters Rutog County, Tibet, China [46]. This path has copious vegetation coverage and is an ideal habitat for many wild animals, such as *Equus Kiang*, *Pantholops hodgsonii*, and *Ovis ammon*. Wild and domestic animals often share environmental resources, such as water and food, which helps facilitate the spread of PPRV between the two populations [47]. Considering that PPRV has been proven to spread between domestic and wild animals, it is crucial to improve the vaccination of domestic animals in this area. Path e starts from Kazakhstan, passes through the junction of the Khorgos and Ili rivers, and finally enters Huocheng County in Xinjiang, China. Huocheng County directly borders Kazakhstan to the west. Animal husbandry is one of the major industries in this area, and cross-border grazing is common in both countries [48]. It has been reported that the PPRV strains from Kazakhstan and China share high similarity with a similarity of 99.8%. This suggests that PPRV has been transmitted between the two countries and that path e is a path of virus transmission [49].

As mentioned previously, livestock density is often correlated with human population density. Therefore, it is likely that human population density is the most important predictive factor because its very nature reflects the distribution of small ruminants. However, it is difficult to obtain high-quality wildlife density data in this area. Due to the complex migration of wild ruminants, it is almost impossible to assess the size of this population accurately. Moreover, the distribution of animals is not uniform and therefore cannot be represented by an average. Besides human population density, it has also been recognized that the behaviors of migration and grazing are also regulated by seasonal environmental factors such as temperature, precipitation, and non-environmental factors. These factors are relatively easy to assess and have all been included in the current MaxEnt models. Considering the high correlation between the prediction results and reported PPRV cases, the current models seem to produce a reliable evaluation of the risk of PPRV.

Large-scale immunization has not been overly successful in preventing the spread of PPRV due to the broad distribution, heterogeneous types, and frequent migration of hosts. There is still no effective prevention and control method for domestic and wild animal diseases in the research area. In 2017, a case of PPRV in endangered saiga in Mongolia attracted global attention and raised the awareness of PPRV among wild animals [50]. PPRV infection in wild animals also limits the ability of global PPR eradication programs to be successful and therefore needs to be addressed [51]. Although our prediction system was constructed to analyze the distribution of PPRV, systematic health surveillance, particularly at the wildlife-livestock interface, must be improved. This study has provided evidence of the origin of PPRV in China and has suggested novel strategies for preventing PPRV transmission across borders to facilitate the global eradication of PPRV as set by the OIE and FAO.

## Supporting information

S1 Data The record of PPR outbreak with latitude and longitude information of the location. (CSV)
